# Why Does Non‐Photosynthetic *Monotropastrum humile* (Ericaceae) Have Scale Leaves?

**DOI:** 10.1002/pei3.70060

**Published:** 2025-06-04

**Authors:** Shiori Harada, Masayuki Shiba, Syuji Kurosu, Hayato Izawa, Kaito Kurotaki, Takato Yasuda, Tatsuya Fukuda

**Affiliations:** ^1^ Graduate School of Integrative Science and Engineering, Tokyo City University Tokyo Japan

**Keywords:** allometry, *Monotropastrum humile*, morphology, mycoheterotrophic plant, non‐photosynthetic, scale leaves

## Abstract

*Monotropastrum humile* (D.Don) H.Hara (Ericaceae), a mycoheterotrophic plant, retains scale leaves of a certain size despite their lack of photosynthetic function. This study aimed to clarify the morphological basis for the persistence of these scale leaves by examining their relationship with floral organs through morphological and anatomical analyses. For the morphometric analysis, measurements were taken at seven locations. For the anatomical analysis, epidermal cells were photographed and analyzed in abaxial and adaxial views. The sizes of scale leaves and floral characters showed allometric growth. *M. humile* is pollinated by long‐tongued bumblebees; it must maintain flower size for effective pollination. Therefore, its scale leaves cannot become allometrically smaller, and it is necessary to invest a large amount of resources into scale leaves. Our studies show that *M. humile* must constrainedly maintain scale leaves to form flowers, even if leaves lose the function of photosynthesis.

## Graphical abstract

The mycoheterotrophic plant *Monotropastrum humile* (D.Don) H.Hara retains non‐photosynthetic scale leaves, which constitute approximately 18% of its total resources. Morphological and anatomical analyses revealed that the scale leaves and flowers exhibit allometric growth, with the plant investing in scale leaves to support flower development. Despite the loss of photosynthetic function, the scale leaves are essential for maintaining flower size, which is crucial for pollination by long‐tongued bumblebees.

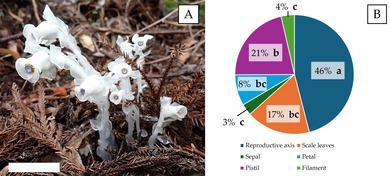

## 1 Introduction

**FIGURE 1:**
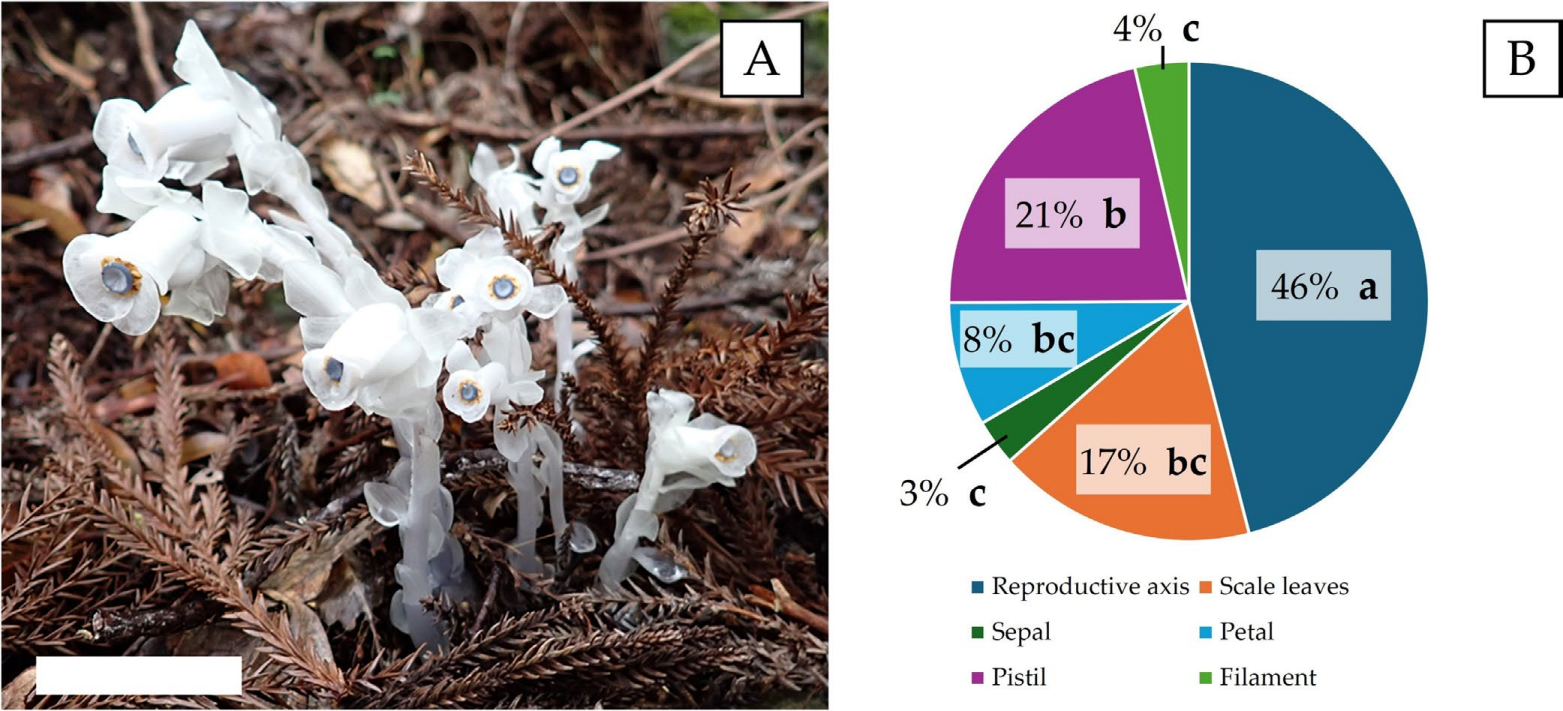
(A) *Monotropastrum humile* (D.Don) H.Hara. Scale bar = 5 cm. (B) Comparison of percentages of character mass to total dry mass. Values in the pie chart labeled with different letters are significantly different according to the Tukey–Kramer test (*p* < 0.05).

Land plants have a wide range of symbiotic relationships with fungi, and the most common symbiotic system is the mycorrhizal symbiotic system, in which plants provide photosynthetic products to fungi and fungi assist plant roots in absorbing inorganic nutrients (e.g., Selosse and Tacon 1998). However, some plants depend on mycorrhizal symbiotic systems for carbon supplementation. In particular, the most extreme mycoheterotrophic plants have lost their photosynthetic ability and obtain carbon necessary for growth entirely via mycorrhizal fungi that inhabit the roots and/or rhizomes of both the host and parasite (e.g., Björkman 1960; Leake 1994). Approximately 500 species of mycoheterotrophic plants are known worldwide (Jacquemyn and Merckx 2019); however, many species exhibit unique morphological and physiological traits that occur during the process of losing their photosynthetic ability, as well as various properties related to their symbiotic relationship with fungi. Therefore, this plant group provides an important research tool for studying the diversification of land plants. Among the mycoheterotrophic plants, genus *Monotropastrum* includes two species *M. humile* (D.Don) H.Hara and *M. kirishimense* Suetsugu, which are widely distributed in Asia (Suetsugu et al. 2023). In *Monotropastrum*, including *M. kirishimense*, it has been observed that each shoot typically bears a single flower (Figure 1A; Suetsugu et al. 2023). *M. humile* roots have a highly specialized association with fungal partners in Russulaceae (Matsuda et al. 2011). Therefore, this species is entirely white, with an erect, unbranched reproductive axis, 5–20 cm tall. Only one flower of *M. humile* is formed at the top of the reproductive axis, pointing slightly downward, and after flowering, it produces white berries (Figure 1A). *M. humile* underwent many morphological and ecological changes during its evolution as a mycoheterotrophic plant, including the loss of leaf laminae and reduced underground vegetative organs (Suetsugu et al. 2023; Freudenstein and Broe 2024). Although the developmental origin of the scale leaves in *M. humile* has not been genetically determined, morphological descriptions in *Flora of Japan* (Takahashi 1993) suggest that they are part of the reproductive axis. However, given their resemblance to papery bracts on the inflorescence rachis reported in other Monotropoideae species (e.g., Rose and Freudenstein 2014), it is possible that these organs represent bract‐like structures rather than reduced vegetative leaves. In this study, we therefore treated the scale leaves as stem‐derived organs. The following questions were raised: Why does *M. humile* have scale leaves even though it does not need to photosynthesize? Are the scale leaves of *M. humile* still undergoing the leaf degeneration process and are yet to completely regress? In fact, our preliminary analysis showed that as much as 17% of *M. humile* aboveground biomass is allocated to the production of scale leaves (Figure 1B). Although no quantitative data on leaf biomass allocation are available for non‐photosynthetic plants, photosynthetic species typically invest approximately 20%–30% of aboveground biomass in their leaves (e.g., Hoeber et al. 2017; Kramer‐Walter and Laughlin 2017; Shiba et al. 2024). Interestingly, *M. humile* appears to invest a comparable or even slightly higher proportion in its scale leaves, despite their lack of photosynthetic function. Hence, the following question arises: Why does *Monotropastrum humile* produce scale leaves? Several possible explanations have been proposed, including protective, structural, or developmental roles. For instance, seed and fruit morphological traits associated with protection and resource investment have been discussed in other mycoheterotrophic species (Suetsugu 2018; Suetsugu et al. 2024), supporting the notion that scale leaves may retain non‐photosynthetic functions. For example, Tsukaya (2018) described a scenario that scale leaves were formed by ectopically expressing floral identity genes in mycoheterotrophic plants. To explore one such possibility, this study aimed to clarify why *M. humile* retains its scale leaves despite their lack of photosynthetic function, from the perspective of allometric growth among organs.

## 2 Materials and Methods

All samples of *M. humile* examined in this study were collected from the Tenryu Branch (N 37′90″; E 137′74″) of Center for education and research field sciences, Faculty of Agriculture, Shizuoka University. Forty‐one individuals were included in this study. Voucher specimens have been deposited in the herbarium of the National Museum of Nature and Science (THS).

**FIGURE 2:**
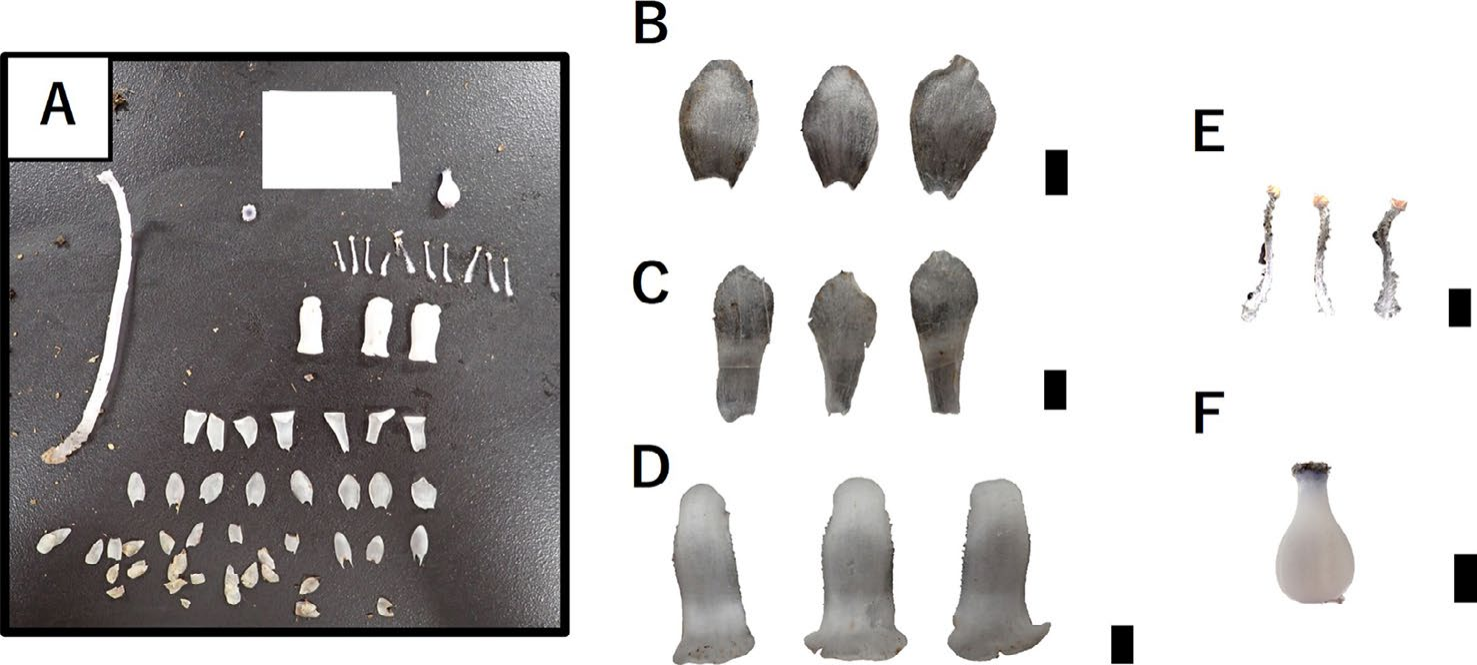
Morphological traits measured in this study. (A) Dissected view of an individual *Monotropastrum humile*, showing each organ separated for measurement purposes. Silhouette of scale leaf (B), sepal (C), petal (D), filament and anther (E), and pistil (including ovary and stigma) (F); scale bars = 5 mm (B–F).

For the morphometric analysis, seven traits were measured (Figure 2). Dimensions such as length, width, and area were recorded for the scale leaves, sepals, and petals (Figure 2B–D). The lengths of the filament and pistil were measured, and the diameter of the stigma apex and ovary was measured for each sample (Figure 2E,F). For each trait, measurements were performed using the image analysis software ImageJ after image acquisition. For the scale leaves, sepals, and petals, filament length, and three organs were measured per individual, and the average value was used for that individual. For the pistil length, stigma apex diameter, and ovary diameter, three measurements were taken from each image, and their average was used as the value for that individual.

For the anatomical analysis, thin sections of scale leaves, sepals, and petals were manually prepared using a razor blade. The outermost surface of each section was mounted in distilled water on a microscope slide and observed under a light microscope. Images of the epidermal cells on both abaxial and adaxial sides were captured using a digital camera system (Moticam1080; SHIMADZU, Japan), and the cell area per epidermal cell was measured using ImageJ.

Plant parts subjected to measurement underwent a drying process at 75°C for a period of 3 days in an incubator (FS‐405; Advantec, Japan). The dry mass of each plant part was measured using an electronic balance (ATC224R; SHIMADZU, Japan). The resource partitioning rate was calculated from the total dry mass of all aboveground organs and the dry mass of each organ.

Statistical analyses were performed using Microsoft Excel for Microsoft 365. After examining the correlation coefficients between the two variables, the coefficient of determination for fitting the linear regression was examined. A multiple comparison test (Tukey–Kramer test, *p* < 0.05) was also performed to compare the three independent groups for area per cell.

## 3 Results and Discussion

**FIGURE 3:**
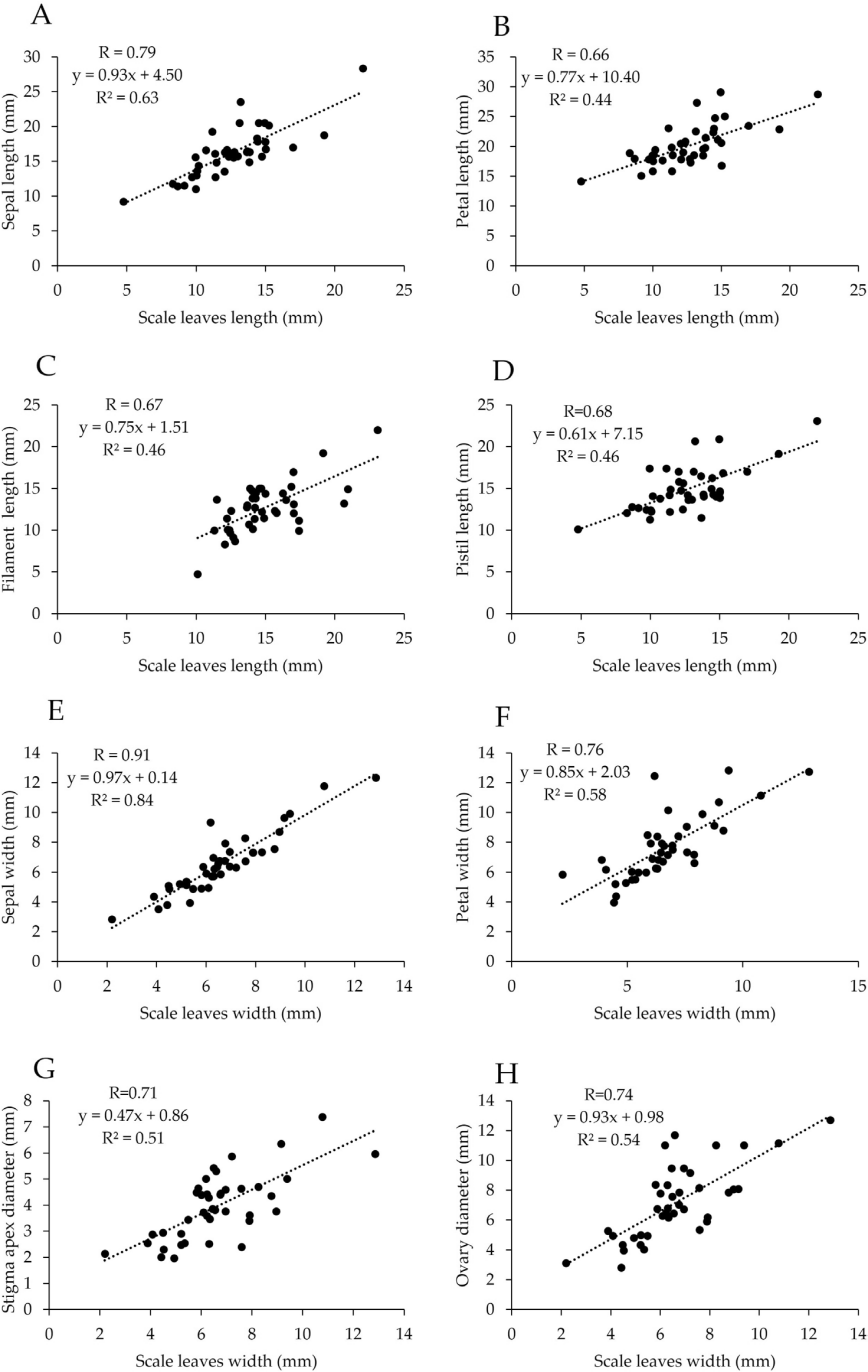
Relationships of length (A–D), width (E–F) between scale leaves and floral characters. (A, E) Sepal. (B, F) Petal. (C, G) Filament. (D, H) Pistil.

**FIGURE 4:**
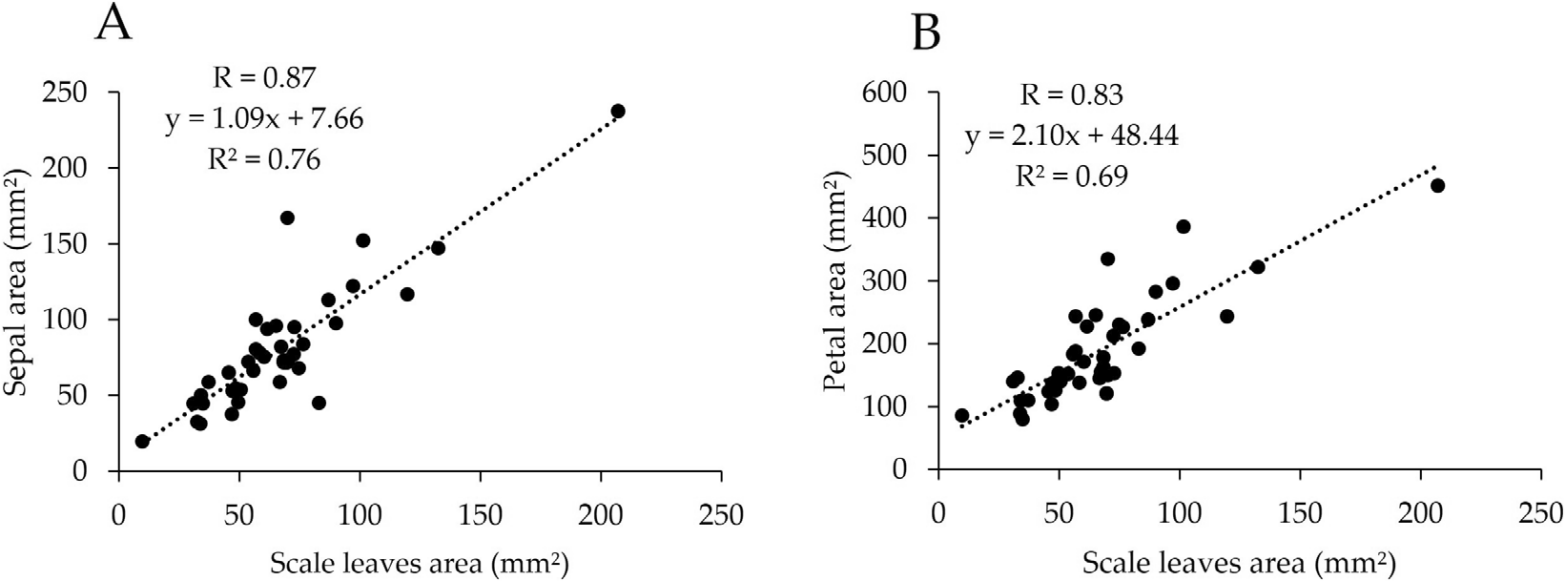
Relationships of area between scale leaves and floral characters. (A) Sepal. (B) Petal.

**FIGURE 5:**
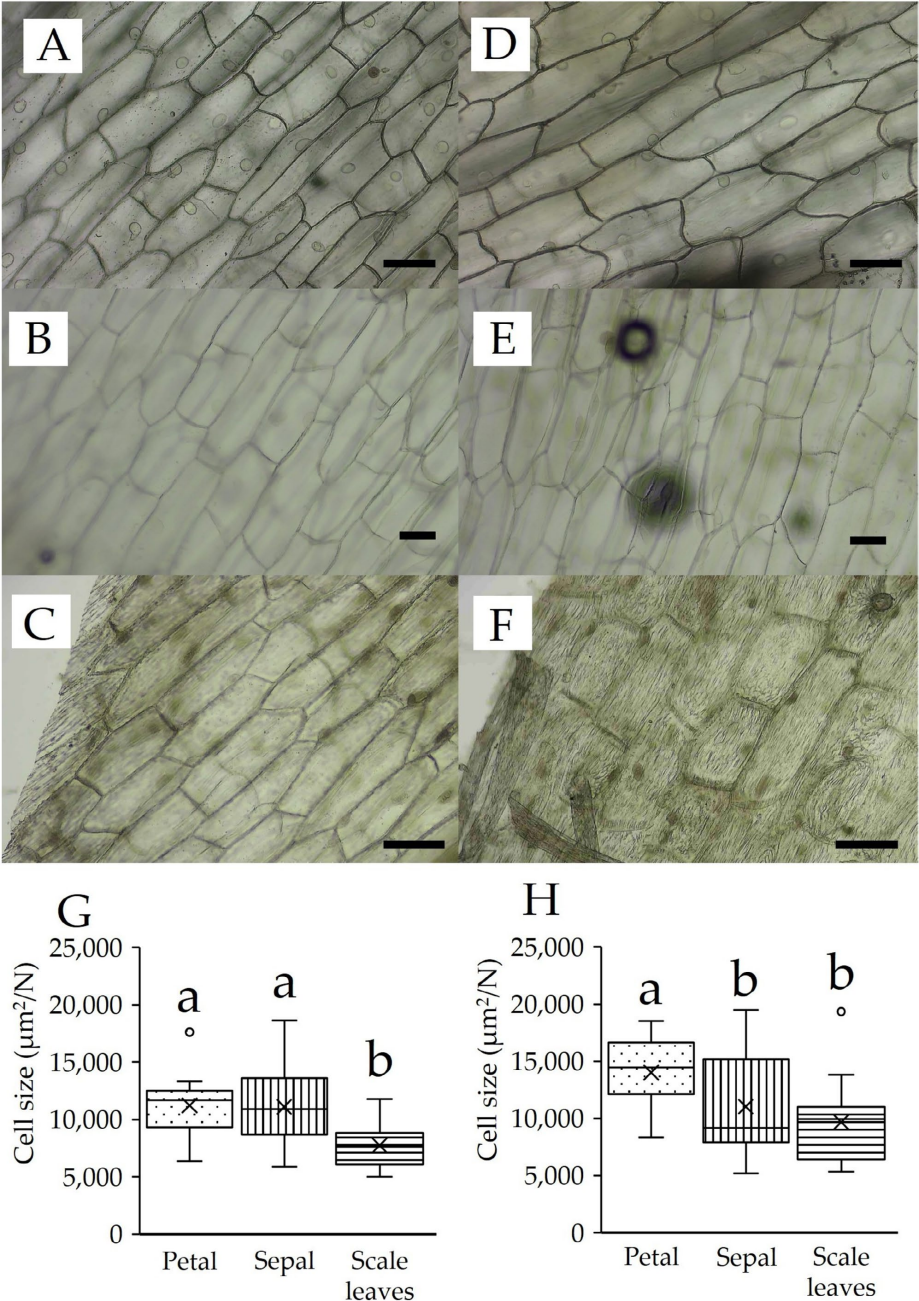
Microscopic images of epidermal cells of a scale leaves (A, D), sepal (B, E) and petal (C, F). (A–C) Abaxial side. (D–F) Adaxial side. Scale bar = 100 mm; comparison of cell size of scale leaves, petals, and sepals. (G) Abaxial side. (H) Adaxial side. Box whisker plots marked with different letters differ significantly according to the Tukey–Kramer test (*p* < 0.05). Crosses indicate the position of the mean.

The dry mass measurements for each part are shown in Figure 1B. These findings suggest that *M. humile* allocates 46% of its biomass to the reproductive axis and 17% to the scale leaves. The remaining biomass was distributed as follows: the ovules received 21% of the biomass, 8% of petals, 4% of filaments, and 3% of sepals. The relationship between the scale leaves and the lengths of other organs is shown in Figure 3A–D. It was linear for all the samples. A positive correlation was observed across all the parts. The correlation coefficient between the scale leaves length vs. sepals length was 0.79, and the coefficient of determination was 0.63 (Figure 3A). The correlation coefficient between scale leaves length vs. petal length was 0.66, and the coefficient of determination was 0.44 (Figure 3B). The correlation coefficient between the scale leaves length vs. filament length was 0.67, and the coefficient of determination was 0.46 (Figure 3C). The correlation coefficient between scale leaves length vs. pistil length was 0.68, and the coefficient of determination was 0.46 (Figure 3D). The relationship between the scale leaves and the widths of other organs is shown in Figure 3E–H. It was linear for all the samples. A strong positive correlation was observed across all the measurement locations. The correlation coefficient between the scale leaves length vs. sepal length was 0.91, and the coefficient of determination was 0.84 (Figure 3E). The correlation coefficient between scale leaves length vs. petal length was 0.76, and the coefficient of determination was 0.58 (Figure 3F). The correlation coefficient between scale leaves length vs. stigma apex diameter was 0.71, and the coefficient of determination was 0.51 (Figure 3G). The correlation coefficient of scale leaves vs. ovary diameter was 0.74, and the coefficient of determination was 0.54 (Figure 3F). The relationship between the scale leaves and the sepal and petal areas is shown in Figure 4. It was linear for all the samples. Strong positive correlations were observed for both the sepal and petal areas. The correlation coefficient between the scale leaves area vs. sepal area was 0.87, and the coefficient of determination was 0.76 (Figure 4A). The correlation coefficient between the scale leaves area vs. petal area was 0.83, and the coefficient of determination was 0.69 (Figure 4B). A comparison of the cell sizes for the scale leaves, sepals, and petals is shown in Figure 5G,H. The results showed that, on the abaxial and adaxial sides, the cell area per cell was the smallest for the scale leaves (mean_abaxial_ = 7743, mean_adaxial_ = 9675), followed by the sepals (mean_abaxial_ = 11,109, mean_adaxial_ = 11,035), and finally the petals (mean_abaxial_ = 11,210, mean_adaxial_ = 14,023).

Allometry is a study of how biological processes such as growth, development, and resource allocation vary with body size and with each other, and how these variations affect ecology and evolution (Niklas 1994). It also describes how the traits of an organism change with size. In fact, various studies have indicated that, in general, the sizes of branches, leaves, flowers, and inflorescences are allometrically related (e.g., White 1983; Primack 1987), which describes the correlated variation in shape and size that could occur within one type of organ or involve relative proportions of different organs. Such allometric growth is characterized by differential growth in different parts of the body (Niklas 1994) and changes in shape with changes in size (Brookstein 1991). For example, the correlation between plant and inflorescence sizes has been attributed to pollinator visits, suggesting that pollinators contact a greater number of flowers in large inflorescences than in small ones (e.g., Donnelly et al. 1998). In addition, Hayakawa et al. (2011) indicated a correlation between the leaf size and floral characteristics of *Cymbidium goeringii* Rchb.f. (Orchidaceae). Thus, the scale leaves of *M. humile* may remain small in size owing to the allometric growth between them and the other organs. In addition, a fundamental feature of multicellular organisms is their ability to coordinate developmental processes at the tissue, organ, and organism levels. Our results indicated significant correlations between certain characters of the analyzed organs (Figures 2 and 3), suggesting that developmental constraints are involved in the correlated evolution of scale leaves, sepals, and petals of *M. humile* , rather than the selection of genes that affect them independently. In general, organ development is mediated by the temporal and spatial regulation of cell proliferation and expansion; therefore, we compared the cell numbers and sizes of these organs (Figure 4). Our results indicated that the cells of *M. humile* grow larger in the order of scale leaves, sepals, and petals (Figure 4), suggesting that some genetic modifications have occurred in the scale leaves. Flower formation in Ericaceae plants follows the ABC model (e.g., Cheon et al. 2017). The ABC model explains how A, B, and C class genes specify floral organ identity at early developmental stages (Coen and Meyerowitz 1991). While sepals are specified by A class genes and petals by A and B class genes, further development involves numerous other genes. Thus, the observed differences in cell size among scale leaves, sepals, and petals in *M. humile* may reflect additional, layered genetic modifications. Moreover, our results showed that the length and width of scale leaves and sepals of *M. humile* were more correlated with external morphology than those of petals and scale leaves (Figures 2 and 3), suggesting that these morphological changes reflected the differences in cells that changed in the order of scale leaves, sepals, and petals. McDonald et al. (2003) and Galen (2006) reported that selection could drive shifts in the sizes of different organs, even if the underlying genes affect each organ independently. Leaves, sepals, and petals are homologous organs that share mechanisms of developmental control (Anastasiou and Lenhard 2007); hence, the genes that act pleiotropically on both organ types might give rise to coordinated changes in shape or size. Virtually all studies on the genetics of leaf and floral development have used mutagenesis to create loss‐of‐function mutations that dramatically alter organ size and shape in some model plants. Using this approach, several major genes that cause gross abnormalities in the leaves and floral development of some plant taxa have been identified and characterized (e.g., Dengler 1984). However, this correlation may not be broadly applicable even among mycoheterotrophic plants, given that leaf reduction in these species often follows distinct developmental trajectories from floral organ formation (Tsukaya 2018). In the future, it will be interesting to analyze the relationship between scale leaves and floral characters of mycoheterotrophic plants, such as *Pyrola aphylla* Sm. (Ericaceae) in the same family and *Cymbidium* species in other families. In addition, our results indicate that if the size of the scale leaf in *M. humile* approaches zero, the size of the floral organ also approaches zero, suggesting that *M. humile* cannot reduce the size of its flowers significantly; hence, it must produce leaves larger than a certain size. However, the closer the size of the scale leaves is to zero, the less investment is made in scale leaves; therefore, it is thought that *M. humile* would be able to invest these resources in other organs and achieve efficient growth. This suggests that floral size in *M. humile* may be subject to a functional constraint, potentially related to its pollination strategy.

Plant–pollinator interactions are one of the main symbiotic relationships between angiosperms and animals and greatly affect plant reproductive success (e.g., Galen 1996). The adaptation of flowers to pollinators is considered an important mechanism leading to the diversification and speciation of angiosperm floral traits (Stebbins 1970; Galen and Newport 1987), and the diversity of floral traits has been recognized as a result of adaptation to pollinators (e.g., Campbell et al. 1997). Floral size, along with other floral traits such as color and structural features, is positively correlated with visual attractiveness to pollinators because it indicates the amount of potential reward (Fenster et al. 2006) and can influence pollen removal and deposition patterns (Rademaker et al. 1997). For *M. humile* , long‐tongued bumblebees such as *Bombus diversus diversus* (Smith) have been reported as likely pollinators in dark understory environments (Tanaka 1978; Kubo and Ono 2014). *B. diversus* shows a preference for larger flowers (Mitchell 1994), and such preference may increase the bee's fitness through improved foraging efficiency (Karron et al. 2004). However, a field study by Ushimaru and Imamura (2002) found no clear evidence that flower size or number significantly enhanced female reproductive success in *M. humile* , suggesting that pollinators may not always respond strongly to floral size. Nevertheless, considering the foraging behavior of *B. diversus* and the need to maintain effective pollinator visitation, *M. humile* may still require flowers above a certain size threshold to ensure successful bumblebee‐mediated pollination. This potential constraint on floral size may also indirectly affect the minimum size of scale leaves, which are developmentally and allometrically linked to floral organs. Therefore, despite not performing photosynthesis, it is conceivable that *M. humile* must invest substantial resources in the formation of scale leaves. We acknowledge the limitation of lacking direct pollinator observations in this study and suggest that future research should investigate scale leaf–floral trait interactions in conjunction with pollinator behavior. Although we conducted no anatomical or histochemical analyses in this study, the presence or absence of vascular bundles, mesophyll tissue, and starch granules in the scale leaves of *M. humile* remains to be determined. Clarifying these structural features will be important for understanding the functional role and evolutionary reduction of scale leaves in mycoheterotrophic plants.

## Conflicts of Interest

The authors declare no conflicts of interest.

## Supporting information


**Table S1.** Dry mass measurements of each part.

**Table S2.** Length measurement results for each part.

**Table S3.** Width measurement results for each part.

**Table S4.** Measurements of the area of each part.

**Table S5.** Measurements of cell sizes of each part.

## Data Availability

Data available in article Tables [Link], [Link].
